# Next-generation antenna beamforming via caterpillar fungus optimization for enhanced wireless communication

**DOI:** 10.1038/s41598-026-55840-y

**Published:** 2026-06-30

**Authors:** Samar I. Farghaly, Mona Gafar, Abdullah M. Shaheen, Ahmed S. Alwakeel, Shadia Sarhan

**Affiliations:** 1 Department of Electronics and Electrical Communications Engineering, Faculty of Engineering, Tanta University, Tanta, 31111 Egypt; 2 Department of Computer Engineering and Information, College of Engineering, Wadi Ad Dwaser, Prince Sattam Bin Abdulaziz University, 16278 Al-Kharj, Saudi Arabia; 3 Machine Learning and Information Retrieval Department, Artificial Intelligence, Kafrelsheikh University, Kafrelsheikh, 33516 Egypt; 4 Department of Electrical Engineering, Faculty of Engineering, Suez University, P.O. Box: 43221, Suez, Egypt; 5 Faculty of Computers and Artificial Intelligence, Benha University, Benha, 13511 Egypt

**Keywords:** Beamforming, Antenna array synthesis, Caterpillar fungus optimization, Side lobe levels, MIMO, Uniform linear array., Engineering, Mathematics and computing

## Abstract

Beamforming has emerged as an essential enabling technique for beyond 5G and future 6G systems because it improves spectral efficiency. However, optimizing antenna weights in beamforming is a highly nonlinear and multidimensional problem that traditional approaches struggle to solve. To address this, we offer a unique beamforming strategy based on the Caterpillar Fungus Optimization (CFO) algorithm, which strikes an optimal balance between exploration and exploitation, making it ideal for large-scale antenna systems. The CFO is inspired by the rare lifecycle of caterpillar fungus considering its soil exploration and parasitic behaviors. Its unique blend of wave-like and spiral search strategies, dual parasitism operators, and hybrid noise-handling makes it stand out among bio-inspired algorithms, enabling high accuracy and robustness in complex engineering optimization problems. The proposed scheme has two goals: first, to reduce the number of active antenna elements, thereby improving energy efficiency and reducing system complexity; and second, to suppress side lobe levels (SLL), which mitigate interference and improve communication performance. To assess its efficacy, the CFO-based method is compared to five established algorithms: Artificial Rabbits Optimizer (ARO), Whale Shark Optimization (WSO), Grey Wolf Optimizer (GWO), Particle Swarm Optimization (PSO), and Boomerang Aerodynamic Ellipse (BAE). According to simulation data, CFO maintains beamwidth deviations within 1% to 2% of the standard reference while achieving an average error reduction of up to 99.7% when compared to PSO and WSO. Furthermore, CFO outperforms all benchmark algorithms in terms of accuracy, and computing efficiency, delivering the lowest SLL deviations and the most steady convergence behavior. This paper provides a simulation-based beamforming optimization framework that employs the metaheuristic Caterpillar Fungus Optimization (CFO) algorithm for antenna array synthesis in beyond 5G and future 6G wireless systems.

## Introduction

In recent years, radio communication systems have observed huge growth in the number of users and bandwidth-intensive applications, creating a considerable demand for high-capacity wireless communication. Fifth-generation (5G) and emerging sixth-generation (6G) mobile networks satisfy this demand by operating in high-frequency bands, allowing for multi-gigabit-per-second (Gbps) data rates and larger bandwidth than conventional frequency bands^1^. As a result, millimeter-wave communications has received substantial research attention as a crucial technology for next-generation wireless networks^2,3^. However, millimeter-wave systems encounter a number of propagation issues despite their huge bandwidth availability, such as significant path loss, strict directivity requirements, and blockage sensitivity because of the signals’ small wavelength^1^. Massive multiple-input multiple-output (MIMO) systems combined with beamforming techniques are generally regarded as an efficient way to get around these restrictions and preserve dependable connections between users and base stations^4–6^. Beamforming employs antenna arrays and spatial filtering using angle of arrival (AoA) information^7–9^ to send or receive signals in desired directions. When compared to omnidirectional operation, this directed transmission greatly improves transmit and receive gains^10,11^. Therefore, by boosting signal power toward intended users while lowering radiation toward undesirable directions, beamforming can compensate for propagation and penetration losses in millimeter-wave bands. Phased antenna array configurations in conjunction with electronic beam steering techniques offer such capabilities^12–14^.

Therefore, efforts have been made to develop array antennas with improved gain, directivity, beam steering capability, and lower SLL^15–17^. Furthermore, in order improve antenna directivity, the number of antenna elements must be increased. Thus, Efficient techniques are used to decrease antenna elements while maintaining high gain and directivity^18,19^. These methods construct antenna patterns by controlling amplitudes, phases, excitation placements, and interelement spacing for array synthesis^20,21^.

The relevant literature and the rationale behind the research topic, with the research gap and key contributions, will be covered in the following.

### Related work

Guglielmo Marconi conducted the first beamforming experiments in 1901 when he employed a circular array with four antennas to increase the gain of transatlantic Morse code transmission^22^. Karl Ferdinand Braun, who shared the Nobel Prize in physics with Marconi in 1909 for his contributions to wireless telegraphy, demonstrated in 1905 that a phased array could steer radio waves in a similar early manner^23^. Antenna diversity was created for phased array radars and radio astronomy in the 1940s as a means of overcoming fading^24^. With the advent of phased arrays for sonars in the 1950s and 1960s, antenna arrays could direct signals other than electromagnetic waves^25^. Smart antennas are considered a promising technique for wireless communication systems. It provides robust wireless network solutions that can increase control power and improve capacity, coverage, and service quality^26,27^. Adaptive beamforming (ABF) and the introduction of massive MIMO techniques to increase the capacity of the system are two of the most important and well-known features of the development of smart antenna technology.

The foundation of such methods is the use of various antenna array configurations based on the specific application. These strategies aim to regulate various aspects of the radiation pattern, such as null control and SLL suppression^28–32^. The two most commonly used types of antenna arrays among the different shapes used in real-world systems are the linear antenna array (LAA) and the circular antenna array (CAA). Beamforming optimization for LAA and CAA has been the subject of numerous research works that use various strategies to optimize one or more array characteristics, such as element locations and/or element excitation^33–46^. CAA may steer the beam pattern in any direction, while LAA is the most basic type of antenna array. In practice, though, CAA is more complicated than LAA. A communication system’s energy efficiency can be increased by increasing the beam directivity of its antennas, which calls for more directive antennas. Planar arrays are mostly utilized to obtain more directed and symmetric patterns, making them an interesting research topic for planar antenna arrays (PAA). Its primary areas of use include satellite communications, search and tracking radars, and remote sensing. Numerous research incorporate PAA optimization by regulating individual parameters, such as element excitations or element spacing^47–58^.

Antenna array beamforming is the process of choosing the ideal set of parameters to produce the desired beam pattern. However, because the relationships between these factors are difficult to understand, beam pattern optimizations of antenna arrays become very difficult non-linear issues. Therefore, knowing how to reduce the maximum SLL and enhance antenna array beam patterns is essential. The Taylor synthesis approach^59^, the Chebyshev synthesis method^60^, and the convex optimization method^61^ are a few examples of traditional and classical antenna array SLL suppression techniques. Nonetheless, the growing demands on communication systems suggest that beamforming optimizations utilizing the most modern optimization methods in smart antennas should receive more attention.

Numerous algorithms have been presented, and it has been shown that they are effective in resolving various optimization issues. For example, Harris Hawks Optimization (HHO) has been used to address real-world optimization issues like manufacturing optimization, pattern recognition, power quality, and drug design^62–64^.The Slime Mould Algorithm (SMA) was first presented in^65^ and has since been used in a number of domains to address optimization problems, such as image segmentation, scheduling optimization, and machine learning optimization^66^. Additionally, a number of optimization problems have been resolved using PSO, a well-known and potent population-based metaheuristic^67–70^. Furthermore,^71,72^ described the Runge Kutta Optimizer (RUN) and its uses in many domains. Additionally, algorithm hybridization has been used to increase its efficacy; for instance,^73,74^ use the Hybrid Particle Swarm Optimization with Gravitational Search Algorithm (PSOGSA). The authors of^75^ suggested a novel beamforming method that lowers size and SLL for ULA, Chebyshev arrays, and shaped pattern arrays. They employ a strategy that combines the $L_2$ norm and the GWO. They demonstrated how the proposed GWO approach offered a low-complexity beam pattern that was extremely close to the needed radiation pattern.

Another new optimization technique is CFO which draws its inspiration and unique features directly from the life cycle and behaviors of the Ophiocordyceps sinensis (caterpillar fungus), which infect and consume larvae in high-altitude soils. In nature, the fungus explores soil both horizontally and vertically while searching for host larvae. This inspired two exploration strategies in the algorithm. The first is the wave advance operator that simulates broad, wave-like searching, while the second is the spiral rising operator that mimics upward growth through soil, refining local search^76^. Also, the fungus parasitizes larvae with unique features: one larva per fungus, with possible failed attempts before success. This inspired the algorithm’s dual parasitism strategy. The first is the re-parasitism by retrying with improved solutions to escape local optima, while the second seeks for optimal parasitism for exploiting the best conditions for growth and enhancing convergence speed.

In^77^, Taguchi’s method, a unique approach to global electromagnetic optimization, is used to create a sector beam pattern and a null-controlled pattern. As previously stated, SLL reduction is crucial for communication systems’ interference suppression. The papers^78–85^ are interested in implementing various methods for SLL reduction in this context. The millimeterwave (mmWave) MIMO technology is used as an application due to SLL reduction and the importance of reducing the number of antennas in the wireless communication system^86,87^.

The mmWave produces a large range of viable spectrum bands by dramatically raising the carrier frequency. Furthermore, because operating in the high-frequency band shrinks the size of the antenna, employing microstrip technology enables a large number of antenna components to be packed into a compact space^88,89^. Despite attaining better spectral efficiency (SE) and efficient power usage, the mmWave MIMO system has a high bit error rate and increased system complexity, especially when including a large number of data streams or users. Furthermore, the mmWave system’s entirely digital precoding is expensive and energy-intensive^88^. Therefore, in both single and multiuser contexts, hybrid analog/digital beamforming is the best way to get around the limitations of pure digital or analog beamforming. In addition to using analog phase shifters to accomplish analog beamforming, the system’s complexity with reference to radio frequency chains (RF) can be decreased^90^.

A heterogeneous network (HetNet) supported by a hybrid reconfigurable intelligent surface (H-RIS) was examined by the authors in^91^. They provided a novel approach for maximizing spectral efficiency (SE). Additionally, they suggested a novel approach to optimize the phase shits at the hybrid RIS based on a hybrid PSO-GWO algorithm. In order to maximize the user’s spectral efficiency, the authors in^92^ suggested a novel RIS phase shift design approach that makes use of a GWO. A number of grey wolf search agents were produced by the suggested GWO method. To find the best option, each search agent’s position is adjusted iteratively based on SE performance. The authors in^93^ concentrated on maximizing the downlink sum rate of multiuser systems by optimizing the stacked intelligent metasurfaces (SIM) design. To maximize the system’s sum rate, they modified a GWO for creating SIM phase shifts.

### Motivation of the research problem

The rapid evolution of wireless communication technologies, particularly the transition from 5G to beyond 5G and the anticipated 6G networks, places strict demands on system performance, including increased stability, lower latency, better spectrum efficiency, and larger data rates. In order to meet these needs, beamforming techniques are essential because they reduce interference and signal leakage while directing transmitted signals toward the intended users. However, the optimization of antenna locations, particularly in large-scale antenna systems, is a critical component of beamforming’s efficacy.

Conventional optimization methods commonly encounter this complexity because they are sensitive to initialization, prone to local optima, and computationally expensive in real-time applications. To address these constraints, bio-inspired and nature-inspired metaheuristic algorithms have emerged as effective tools due to their adaptability, resilience, and global search capabilities. The recently introduced CFO algorithm, in particular, has shown promise in tackling nonlinear and multidimensional optimization problems due to its balanced exploration-exploitation mechanism. In addition, CFO applications were validated with outperformance against different well-known metaheuristics (PSO, GWO, EO, HBO) in benchmark and Fuel cell case studies^76^.

Motivated by these considerations, this paper studies the use of the CFO method to optimize beamforming of transmit antennas beyond 5G communication systems. By using CFO’s unique qualities, the goal is to produce considerable increases in system performance parameters such as signal-to-noise ratio, achievable rate, and energy efficiency, addressing the pressing concerns of next-generation wireless networks.

### Research gap and main contribution

Although several metaheuristic algorithms^94,95^, including GWO, PSO, WSO, ARO, and the recently proposed BAE algorithm, have been widely applied to solve complex engineering optimization problems, several limitations persist. Specifically, many of these algorithms suffer from premature convergence and stagnation in local optima when addressing large-scale and highly nonlinear problems. Moreover, their exploration–exploitation balance is often suboptimal, leading to insufficient diversity in the search process and reduced convergence efficiency.

In addition, most existing studies in antenna array optimization and beamforming primarily focus on single-objective optimization, such as sidelobe level (SLL) reduction or array gain enhancement, without jointly considering critical performance metrics like energy efficiency, interference mitigation, and overall system throughput^96^. This limitation restricts their applicability in next-generation wireless systems, where multi-objective optimization and scalability are essential requirements.

Therefore, there is a clear need for a robust and scalable optimization framework that can effectively handle multi-objective beamforming design while maintaining high convergence accuracy and computational efficiency^97^.

To bridge the gap, this research provides a beamforming optimization strategy based on the CFO method. The primary contributions of this work are summarized as follows:

- We present the first implementation of the CFO method to optimize transmit antenna beamforming for networks beyond 5th-generation cellular network (5G), using its superior global exploration and local exploitation balance.
- The proposed approach is tested against well-known algorithms such as ARO^98^, WSO^99^, GWO^100^, PSO^101^, and BAE^102^ to demonstrate its efficacy and superiority in tackling complicated beamforming challenges.
- The technique based on CFO minimizes the number of active antenna elements, resulting in significant energy efficiency and reduced system complexity.
- An SLL suppression technique is incorporated into the CFO architecture to reduce interference and improve the dependability of the communication connection.
- According to the simulation results, CFO outperforms the algorithms considered in terms of system throughput, spectral efficiency, and achievable rates.
- The suggested method offers a scalable and affordable solution, which makes it attractive for practical implementation in wireless networks that go beyond 5G and into the future sixth-generation cellular networks (6G).

The remainder of the paper is organized as follows. The problem formulation is detailed in Section II. Section III provides a discussion of the Caterpillar Fungus Optimization algorithm. Section IV provides numerical findings to check the tightness of the resulting expression, while Section V presents conclusions.

## Problem formulation

**Fig. 1:**
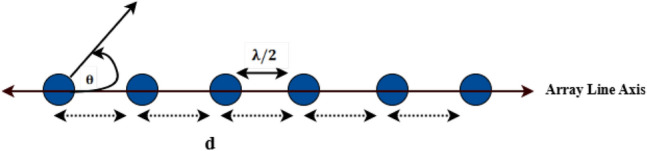
Linear antenna array configuration.

The main objective of this work is to apply the Caterpillar Fungus Optimization (CFO) algorithm to generate linear antenna arrays (LAAs) for both Chebyshev and shaped beam patterns. The optimization procedure seeks to establish the ideal excitation amplitudes and inter-element placements for reproducing the desired radiation pattern with the fewest number of antenna elements possible, hence increasing hardware efficiency and radiation performance. Figure 1 shows the adopted linear antenna array geometry.

For an array consisting of *N* elements, the reference array factor is expressed as

1 $$\begin{aligned} AF_r(\theta )=\sum _{n=1}^{N} I_n e^{j(n-1)\beta d \cos (\theta )}, \end{aligned}$$

where $AF_r(\theta )\in \mathbb {C}^{t \times 1}$ denotes the reference array response evaluated over *t* observation angles, $I_n$ is the excitation coefficient of the $n^{\text {th}}$ element, $\beta =\frac{2\pi }{\lambda }$ is the wave number, $\lambda$ is the wavelength, *d* is the uniform inter-element spacing, and $\theta$ is the observation angle. The excitation vector is written as

2 $$\begin{aligned} \textbf{I}=[I_1,I_2,I_3,\ldots ,I_N]. \end{aligned}$$

For Chebyshev arrays, the array factor can be represented according to whether the number of elements is odd or even. For odd $N=2M+1$,

3 $$\begin{aligned} AF_r(\theta )=I_0+2\sum _{n=1}^{M} I_n \cos (2n\psi ), \quad M=\frac{N-1}{2} \end{aligned}$$

while for even $N=2M$,

4 $$\begin{aligned} AF_r(\theta )=2\sum _{n=1}^{M} I_n \cos [(2n-1)\psi ], \quad M=\frac{N}{2} \end{aligned}$$

where

5 $$\begin{aligned} \psi =\frac{\beta d \cos \theta }{2}. \end{aligned}$$

The compact Chebyshev representation is given by

6 $$\begin{aligned} AF_r(\theta )=T_N\left( \cos \frac{\psi }{2}\right) , \end{aligned}$$

where $T_N(\cdot )$ denotes the Chebyshev polynomial of order *N*.

For the synthesized nonuniform array containing *R* active elements, the array factor is defined as

7 $$\begin{aligned} AF_n(\theta )=\sum _{r=1}^{R} w_r e^{j(r-1)\beta d_r \cos (\theta )}, \end{aligned}$$

where $w_r$ is the excitation weight of the* r*th synthesized element and is written as

8 $$\begin{aligned} \textbf{W}=[w_1,w_2,w_3,\ldots ,w_R], \end{aligned}$$

while $d_r$ denotes the position of the * r*th element in the nonuniform array, determined recursively as

9 $$\begin{aligned} d_r=d_{r-1}+\Delta d_r, \quad r=1,2,\ldots ,R \end{aligned}$$

where $\Delta d_r$ is the spacing increment between adjacent synthesized elements.

The optimization objective is to make the synthesized pattern $AF_n(\theta )$ closely match the required pattern $AF_r(\theta )$ while preserving the half-power beamwidth (HPBW) and reducing the number of active elements. Accordingly, the optimization problem is formulated as

10a $$\begin{aligned} \min F&= \sum _{\theta \in \Omega }\left| AF_n(\theta )-AF_r(\theta )\right| ^2 \end{aligned}$$

10b $$\begin{aligned} \text {s.t.}\quad HPBW_n&= HPBW_r \end{aligned}$$

10c $$\begin{aligned} w_r^{\min }&\le w_r \le w_r^{\max }, \quad r=1,2,\ldots ,R \end{aligned}$$

10d $$\begin{aligned} d_r^{\min }&\le d_r \le d_r^{\max }, \quad r=1,2,\ldots ,R \end{aligned}$$

where $\Omega$ denotes the sampled angular observation region, $HPBW_r$ and $HPBW_n$ are the half-power beamwidths of the reference and synthesized patterns, respectively.

Therefore, each candidate solution handled by CFO is represented as

11 $$\begin{aligned} X=[w_1,w_2,\ldots ,w_R,d_1,d_2,\ldots ,d_R], \end{aligned}$$

and the optimizer iteratively updates this decision vector until the minimum objective value is achieved.

## Caterpillar fungus optimization algorithm

In the CFO optimization process, each candidate solution is encoded as a vector comprising the antenna excitation amplitudes and/or inter-element positions based on the synthesis case under consideration. The fitness of each candidate is evaluated using the objective function provided in Eq. (10), which minimizes pattern mismatch error. Simultaneously, the operational constrains must be achieved by maintaining the required HPBW. To enforce this requirement, the constraint $HPBW_r = HPBW_n$ is addressed through a penalty-based handling approach. Consequentially, any difference between the synthesized and needed beamwidths is augmented to the fitness function as a weighted penalty. As a result, candidate solutions that break the HPBW condition obtain higher objective values and are less likely to survive the optimization process. This technique enables CFO to minimize sidelobe levels, reduce pattern error, and maintain beamwidth without requiring an explicit repair stage.

In the CFO, each search agent represents one ascospore/individual fungus searching for a larva. The CFO depends on a random initialization mimics spores distributed across a soil layer where the fungus population initially explores many microhabitats (step 1). The CFO models the fungus horizontal and vertical exploration of soil to find host larvae via the wave advance operator and the spiral rising operator (step 2). Added to that, the CFO models a dual parasitism strategy considering re-parasitism and optimal parasitism (step 3), where each fungus targets one larva, potentially after several unsuccessful attempts. After that, an assessment is implemented for each individual fungus agent by evaluating the fitness function where the best search agent is recorded and updated in each iteration. These steps are repeated until the maximum number of iterations is maintained. These steps are described and mathematically modeled as follows:

### Step 1: Initialization

Considering a population size of *N*, and maximum iterations of $Max_{iter}$, each candidate solution (fungus position) can be represented as follows:

12 $$\begin{aligned} X_{CF,i} = [X_{CF,1}, X_{CF,2}, . . .,X_{CF,Dim}], \,\,\, i=1:N \end{aligned}$$

13 $$\begin{aligned} X_{CF,i}^{T=0} = Lp_k + z_1 \times (Up_k - Lp_k), \,\,\, i=1:N, k=1:Dim \end{aligned}$$

where, $Lp_k$ and $Up_k$ represent the lower and upper limits of the *k*-th decision variable, while $z_1$ is a uniformly distributed random vector between 0 and 1. Following that, each fungus’s fitness ($F_i$) is computed, and the best solution $X_{CF,best}$ is recorded.

### Step 2: exploration phase in CFO

Each fungus searches soil in wave-like and spiral-like manners. Therefore, two operators are applied with equal probability (50%). First, the wave advance operator is applied as follows:

14 $$\begin{aligned} X_{CF,i}^{T+1} = {\left\{ \begin{array}{ll} X_{CF,i}^{T} + (\alpha - z_2) \times (X_{CF,best} - X_{CF,i}^T) & if \,\, i=1 \\ X_{CF,i}^{T} - z_2 \times (X_{CF,i-1}^T - X_{CF,i}^T) + \alpha \times (X_{CF,best} - X_{CF,i}^T) & Else \end{array}\right. } \end{aligned}$$

15 $$\begin{aligned} \alpha = 2 \times z_3 \times cos(\pi z_3) \end{aligned}$$

where, $z_2$ and $z_3$ are random factors following uniform distribution (*U*(0,1)). Second, the spiral rising operator can be applied as follows:

16 $$\begin{aligned} X_{CF,i}^{T+1} = {\left\{ \begin{array}{ll} X_{CF,i}^{T} + (\beta - z_4) \times (X_{CF,best} - X_{CF,i}^T) & if \,\, i=1 \\ X_{CF,i}^{T} - z_4 \times (X_{CF,i-1}^T - X_{CF,i}^T) + \beta \times (X_{CF,best} - X_{CF,i}^T) & Else \end{array}\right. } \end{aligned}$$

17 $$\begin{aligned} \beta = 2 \times (\frac{T}{Max_T})^{z_5 z_6} \times cos(\pi z_5) \end{aligned}$$

where $z_4$ and $z_5$ are random factors following uniform distribution (*U*(0,1)) while *T* denotes the current iteration number and $Max_T$ is the maximum allowed number of iterations and their proportion term ($\frac{T}{Max_T}$) controls step size shrinkage, enabling transition from exploration to exploitation. Furthermore, $z_6 \in \{1,2\}$ is a randomly selected integer variable.

### Step 3: parasitism phase in CFO

Inspired by fungus parasitizing larvae, two strategies are represented including the re-parasitic behavior modeled in Eq. (18) to support global diversification, and the optimal parasitic behavior modeled in Eq. (19) to support the local intensification:

18 $$\begin{aligned} X_{CF,i}^{T+1} = X_{CF,i}^T + 3 \times z_7 \times ( (z_8 \times X_{CF,best}) - (z_9 \times X_{CF,i}^T) ) \end{aligned}$$

19 $$\begin{aligned} X_{CF,i}^{T+1} = X_{CF,best} + 3 \times \lambda \times ( (z_7 \times X_{CF,i-1}^T) - (z_8 \times X_{CF,i}^T) ) \end{aligned}$$

20 $$\begin{aligned} \lambda = z_5 \times ( (\frac{T}{Max_T})^2 - 2 \times (\frac{T}{Max_T}) + 1 ) \end{aligned}$$

where $z_7$, $z_8$ and $z_9$ are random factors following uniform distribution (*U*(0,1)).

Therefore, the re-parasitic behavior via Eq. (18) produces small perturbations most of the time, but occasionally large jumps to help escape local optima. On the other side, optimal parasitic behavior via Eq. (19) ensures large exploration in early iterations at large $\lambda$ and fine exploitation later as it reduces approaching to zero at the final iterations count. In the CFO, either the re-parasitic behavior or the optimal parasitic behavior is activated for each fungus’s solution with 50% probability.

### Step 4: fitness evaluation and updating

After updating each of fungus’s seeking solution, the boundary limits are reviewed to ensure that every variable remains within the allowable search range. This approach avoids out-of-range solutions by pushing any surpassing variables back into permitted ranges.

21 $$\begin{aligned} X_{CF,i,k}^{T+1} = min ( max( (X_{CF,i,k}^{T+1},Lp_k) , Up_k ) ); i=1:N, k=1:Dim \end{aligned}$$

For each fungus’s candidate, the fitness function is computed, and the best solution is updated if the candidate achieves a better solution than the current global best as follows:

22 $$\begin{aligned} X_{CF,best} = {\left\{ \begin{array}{ll} X_{CF,i}^{T+1} & if \,\, f(X_{CF,i}^{T+1}) \le f(X_{CF,best}) \\ X_{CF,best} & Otherwise \end{array}\right. };i=1:N \end{aligned}$$

**Fig. 2:**
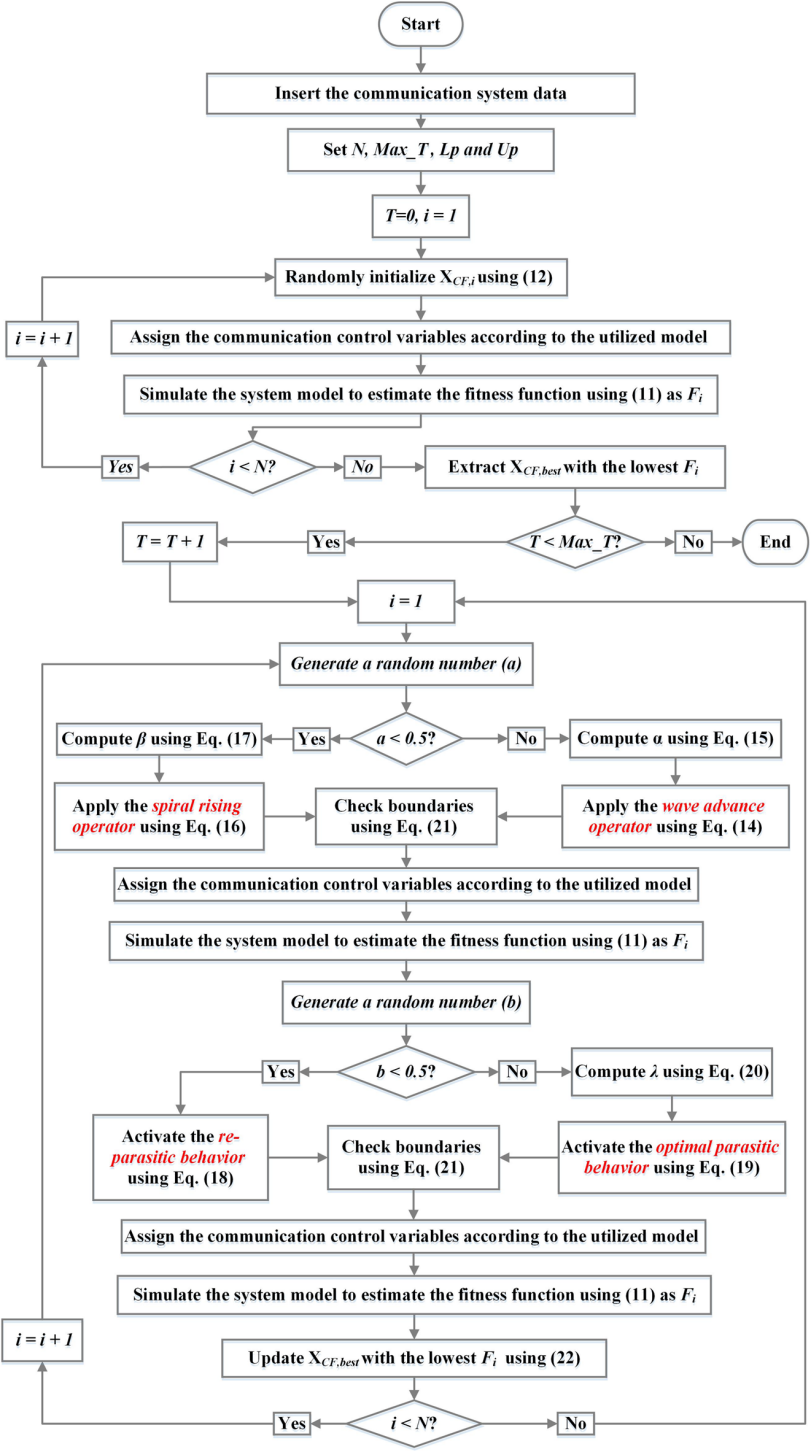
Flow chart of the developed CFO algorithm.

Figure 2 depicts the primary phases of the CFO.

To further clarify the implementation details of the CFO algorithm, Algorithm 1 presents the corresponding pseudocode based on the workflow illustrated in Fig. 2.

**Algorithm 1:**
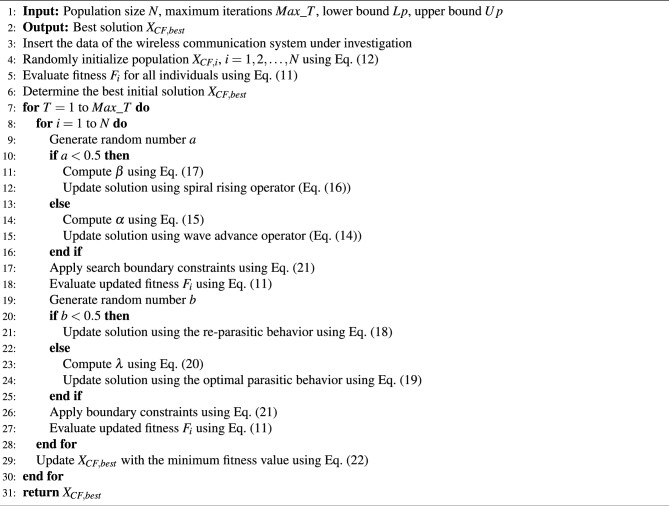
Caterpillar fungus optimization (CFO)

##  Numerical results

This section details MATLAB simulations conducted to validate the effectiveness of the Caterpillar algorithm in reducing the number of antenna elements in a synthesized LAA. Furthermore, the practical applicability of these methodologies is confirmed through validation using computer simulation technology (CST) the Microwave Studio software package, specifically employing a half-wavelength dipole element. The $\lambda /2$ dipole is 135 mm in length and 5 mm in radius with a gap of 20 mm. Furthermore, the array of dipoles is placed at the y-axis and oriented to the z-axis. Consequently, the results of the synthesized array are discussed as follows:

Subsection   “Synthesizing of Chebyshev pattern” presents the proposed result pattern for synthesizing the Chebyshev pattern and compares it with other optimization algorithms. While “Synthesizing of shaped pattern” performs the proposed result pattern of synthesizing the shaped pattern. Finally, Subsection “Statistical outcomes” presents the statistical results of the proposed optimization algorithm CFO. Now, let us begin by explaining them in detail.

Table 1 summarizes parameter settings for all comparison optimization algorithms utilized in this study. To guarantee a fair and unbiased comparison, each technique was run with the same population size, maximum number of iterations, random uniform initialization within the search bounds, and stopping criterion.

**Table 1:** Parameters setting of the applied algorithms.

Algorithms	Parameter setting
ARO	Number of artificial rabbits = 50 Maximum number of iteration = 200
GWO	Number of gray wolfs = 50 Maximum number of iteration = 200
PSO	Number of particles = 50 Maximum number of iteration = 200 c1 = 2; % Cognitive parameter c2 = 2; % Social parameter
WSO	Number of Whale Sharks = 50 Maximum number of iteration = 200 fmax=0.75; % Maximum frequency of the wavy motion fmin=0.07; % Minimum frequency of the wavy motion tau=4.11
BAE	Number of Boomerangs = 50 Maximum number of iteration = 200 Beta parameter = 0.5 Alpha parameter is increasing iteratively
CFO	Number of Caterpillars = 50 Maximum number of iteration = 200

*Initialization methods*: All applied algorithms are randomly initialized using uniform distribution within the search boundaries.

*Stopping criterion*: All applied algorithms are stopped when the maximum number of iterations are reached.

To evaluate optimization performance, MATLAB simulations were run, and electromagnetic validation was performed using CST Microwave Studio. A 135 mm long half-wavelength dipole antenna with a radius of 5 mm and a feed gap of 20 mm was employed. The array elements were placed along the y-axis and aligned with the z-axis. The optimization process was carried out across a predetermined maximum number of 200 separate runs.

### Performance metrics

To ensure clarity and consistency, the performance metrics utilized in this study are described below. The optimization error is calculated as the discrepancy between the synthesized array factor and the desired reference pattern over the sampled angular region, expressed as

23 $$\begin{aligned} Error=\sum _{\theta \in \Omega }\left| AF_n(\theta )-AF_r(\theta )\right| ^2 . \end{aligned}$$

The side lobe level (SLL) is the greatest sidelobe magnitude outside the main-lobe zone, expressed in decibels relative to the main beam peak. The half-power beamwidth (HPBW) is the angular width between the two directions at which the radiated power drops to half of its peak value (i.e., $-3$ decibels). The beamwidth deviation is calculated as the absolute difference between the synthesised and required beamwidths, given by

24 $$\begin{aligned} \Delta HPBW = |HPBW_n - HPBW_r|. \end{aligned}$$

The lowest objective function value attained after the greatest number of iterations is referred to as the convergence accuracy. Furthermore, any reported percentage improvement is computed in relation to the benchmark procedure using

25 $$\begin{aligned} Improvement(\%)=\frac{F_{benchmark}-F_{CFO}}{F_{benchmark}}\times 100, \end{aligned}$$

where *F* represents the corresponding fitness or error value.

### Synthesizing of Chebyshev pattern

In this subsection, we discuss the results of synthesizing Chebyshev LAA. Our objective is to minimize the number of antenna elements while simultaneously preserving the gain of the LAA and SLL. Furthermore, we aim to maintain the lowest possible values for both dynamic range ratio (DRR) and least mean square error (LMSE). The standard parameters of the Chebyshev arrays used in the comparison are $N=20$ elements $SLL=-29.98~dB$, inter-element spacing $d=0.5 \lambda$, and the half power beam width (HPBW) = $6.1879^o$

#### Matlab simulation

**Fig. 3:**
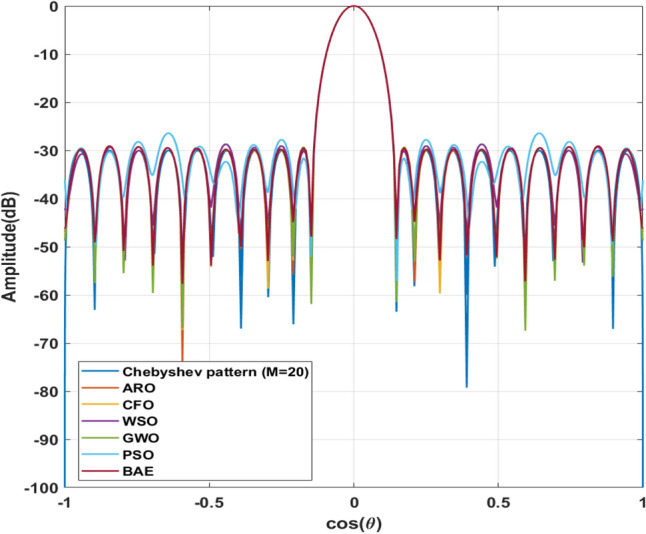
Synthesized LAA Chebyshev pattern with $N=12$ elements.

**Fig. 4:**
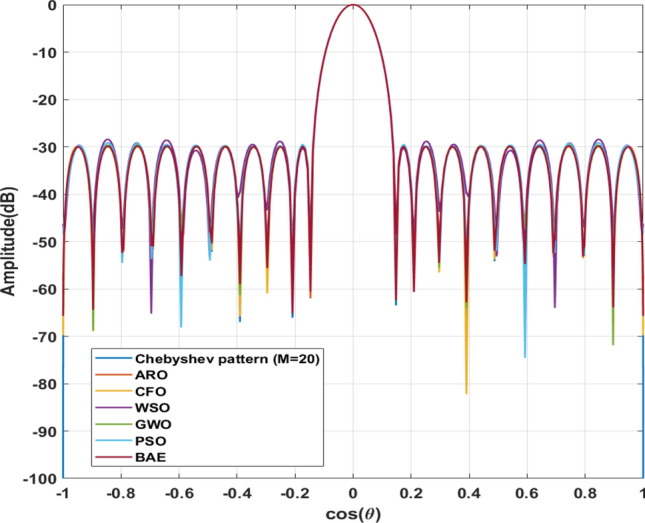
Synthesized LAA Chebyshev pattern with $N=13$ elements.

Figures 3 and 4 illustrate the outcomes of synthesizing a Chebyshev array. These results compare the proposed “caterpillar” optimization algorithm against other optimization algorithms, including ARO, WSO, GWO, PSO, and the BBAE, against the theoretical Chebyshev pattern, thereby demonstrating its superior effectiveness. Figure 3 shows the result of using only $N=12$ elements, while Fig. 4 is the result of using $N=13$ elements. Using a lesser number of antenna elements achieves savings of about $40 \%$ and $35\%$ in hardware. This will reduce the system complexity maintaining the same gain. Observing the main lobe, all algorithms successfully converge to the desired peak amplitude at $cos(\theta ) = 0$, indicating proper beam steering and maximum radiation in the desired direction. In addition, $HPBW=6.1879^o$ closely matches the theoretical Chebyshev pattern. The critical aspect for comparison lies in sidelobe reduction. The proposed optimization algorithm demonstrates good adherence to SLL . Specifically, the Caterpillar algorithm generally aligns very closely with the Chebyshev pattern, particularly in the side-lobe regions. This indicates its effectiveness in achieving the desired SLL characteristics and suppression of interference. where $SLL=-29.07$ at $N=12$ elements, while $SLL=-29.9$ at $N=13$ elements. Tables 3, 4, 7 Summarize the comparison between the proposed algorithm and the other in comparison.

#### CST simulation

**Fig. 5:**
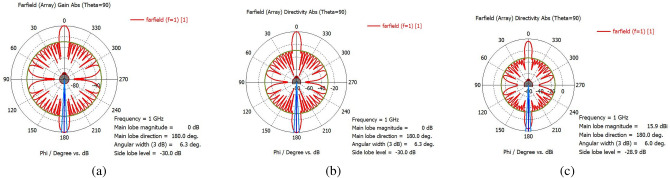
Field pattern of the Chebyshev array using CST with $N=20$ in (**a**), and synthesized patterns with $N=13$ and $N=12$ in (**b**) and (**c**), respectively.

**Fig. 6:**
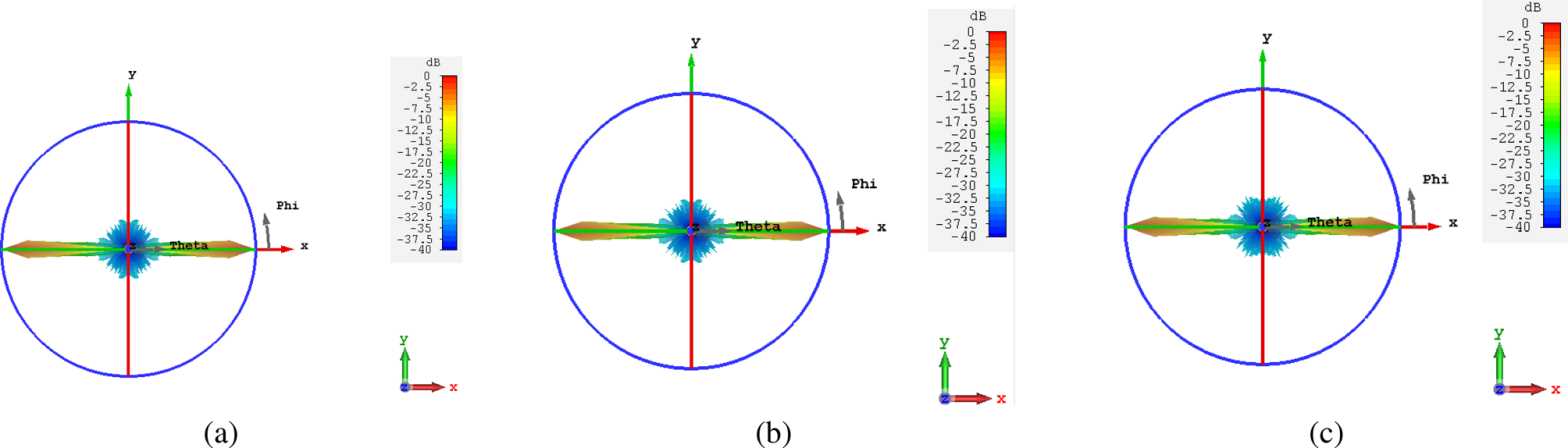
3D pattern of the Chebyshev array using CST with $N=20$ in (**a**), and synthesized patterns with $N=13$ and $N=12$ in (**b**) and (**c**), respectively.

Figures 5 and 6 present the normalized field pattern of the Chebyshev array in $\theta =90^o$ plane and its normalized 3-D pattern. These figures compare the standard Chebyshev array with $N=20$ elements and the CFO algorithm with $N=12, 13$ elements. From the comparison, the proposed algorithm matches well the required pattern. The SLL and HPBW of CFO are exactly identical to those of the standard where the SLL is equal to $-30~dB$ and $HPBW=6.3^o$ by using 13 elements. While the SLL is equal to $-28.9~dB$ and ${HPBW}=6^o$ by using 12 elements, which is excellent result

### Synthesizing of shaped pattern

#### Matlab simulation

**Fig. 7:**
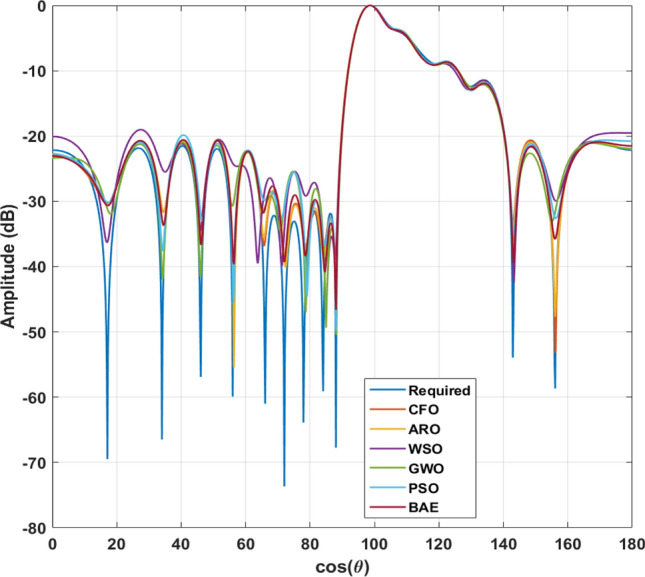
Synthesized LAA shaped pattern with $N=12$ elements.

**Fig. 8:**
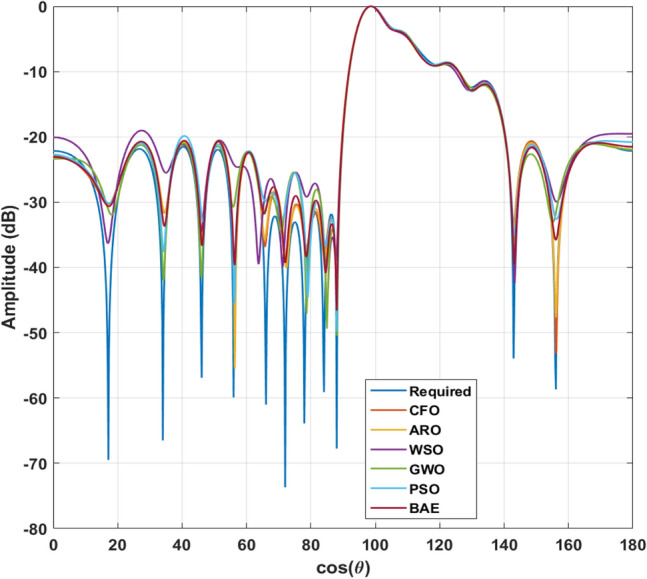
Synthesized LAA shaped pattern with $N=13$ elements.

Figures 7 and  8 illustrate the performance of the proposed algorithm (with $N=12$ and $N=13$) against a shaped beam pattern with $N=16$ and other optimization algorithms for comparison. The CFO produces a pattern that closely matches the desired array pattern, with their excitation coefficients listed in Table 7. Further analysis in Fig. 7 shows that synthesizing the shaped beam pattern with just 12 elements highlights the superiority of the CFO approach over the other technique, achieving a $25\%$ reduction in the total number of array elements. Tables 5, 6, 7 , and 8 provide the location and excitation coefficients of the elements. As shown in Table 9, these findings confirm that the CFO significantly improves performance compared to the other optimization algorithm and ultimately delivers the best results.

#### CST simulation

**Fig. 9:**
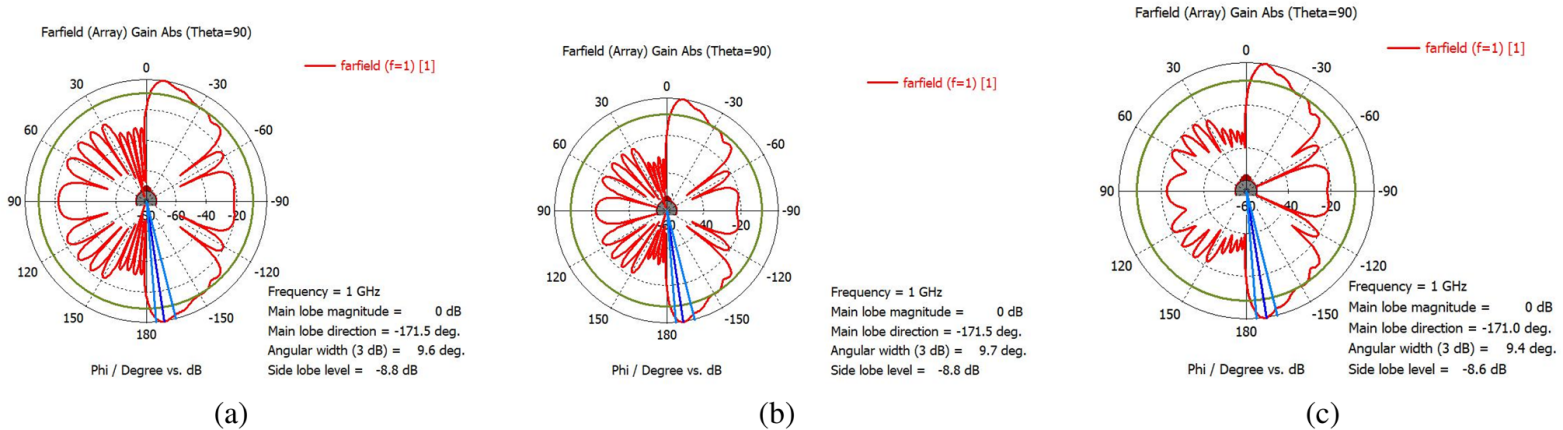
Field pattern of the shaped array using CST with $N=16$ in (**a**), and synthesized patterns with $N=13$ and $N=12$ in (**b**) and (**c**), respectively.

**Fig. 10:**
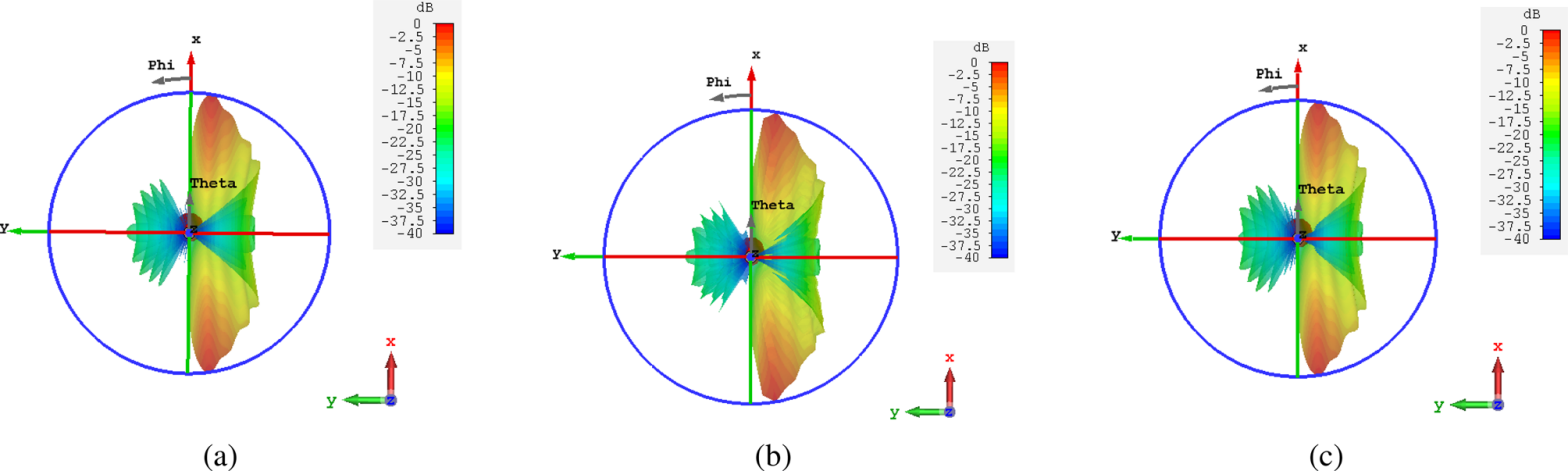
3D pattern of the shaped array using CST with $N=16$ in (**a**), and synthesized patterns with $N=13$ and $N=12$ in (**b**) and (**c**), respectively.

In this part, the normalized field pattern of the shaped array in the $\theta =90^o$ plane and its normalized 3-D pattern are displayed in Figs. 9 and 10. These figures compare the shaped pattern with $N=16$ elements and the CFO algorithm with $N=12, 13$ elements. From the comparison, the proposed algorithm matches well the required pattern. The SLL and HPBW of CFO are exactly identical to those of the standard where the SLL is equal to $-8.8~dB$ and ${HPBW}=9.6^o$ by using 13 elements. While the SLL is equal to $-8.6~dB$ and ${HPBW}=9.4^o$ by using 12 elements.

The obtained radiation pattern data clearly indicate the CFO algorithm’s efficacy in suppressing sidelobes while preserving main lobes. The synthesized patterns generated by CFO closely follow the necessary reference responses while retaining a well-defined primary beam. The lower sidelobe levels indicate better interference rejection and less unwanted power leakage toward non-target directions, which is especially relevant in congested wireless communication scenarios. Simultaneously, the close agreement between the synthesized and reference main-lobe widths supports the precise preservation of the HPBW, ensuring that the required coverage region and directional resolution are not compromised. These results demonstrate that CFO successfully balances radiation quality and pattern fidelity while minimizing unwanted beam response distortions.

### Statistical outcomes

To evaluate the stability, robustness, and convergence dependability of the optimization algorithms, it is crucial to examine their statistical behavior in addition to radiation patterns and element reduction. A thorough statistical comparison of the suggested CFO algorithm with benchmark techniques, such as ARO, GWO, PSO, WSO, and BAE, is provided in this paper. Key statistical indicators, including the minimum, maximum, average, and standard deviation of the objective function values, are reported after the analysis is conducted over several independent runs. These measures offer more in-depth information about each algorithm’s consistency, showing not just its peak performance but also its capacity to produce consistent outcomes throughout several simulations. To further illustrate the superiority of CFO in attaining precision and stability for antenna array synthesis, convergence patterns and distribution features are investigated.

#### Error performance evaluation of CFO and benchmark algorithms

Table 2 compares the error performance ($error = (AF_n - AF_r)^2$) of the CFO approach to ARO, GWO, PSO, WSO, and BAE in Chebyshev and shaped array beam patterns. The results demonstrate CFO’s superior precision and durability.

For the Chebyshev array with $N=12$, CFO has the lowest average error (6.04) and a minimal standard deviation (0.0028). When compared to ARO (7.23) and GWO (14.11), CFO reduces average error by approximately 16.5% and 57.2%, respectively. The improvements are more dramatic against PSO (303.9), WSO (169.8), and BAE (3.52$\times$ $10^7$), with reductions of 98.0%, 96.4%, and nearly 100%, respectively, emphasizing CFO’s reliability and stability.

Increasing to a Chebyshev array with $N=13$, CFO obtains an average error of 0.42, which is lower than ARO by 76.6%, GWO by 93.1%, PSO by 99.7%, WSO by 98.5%, and BAE by 99.7%. Additionally, the maximum error is significantly decreased, and CFO performs significantly better than all of its rivals.

CFO records an average error of 3.37 for the shaped array with $N=12$, which is almost the same as ARO’s 3.37. However, the STD is reduced by 59.2%, indicating more consistent performance. CFO achieves improvements of 49.7% and 81.5%, respectively, in comparison to GWO (6.70) and WSO (18.24). Similarly, the reductions are 73.2% and 67.7%, respectively, in comparison to PSO (12.59) and BAE (10.44).

Finally, CFO attains an average error of 1.78 in the shaped array with $N=13$, which is 73.6% lower than BAE, 80.9% lower than WSO, and 72.7% lower than PSO. In this case, CFO gives the lowest minimum error (0.055) and exhibits strong stability, exceeding others in terms of consistency, while GWO and ARO obtain somewhat lower averages.

In general, CFO not only produces the most accurate results but also shows significant percentage gains over benchmark algorithms, with error reductions anywhere from 16% to over 99% depending on the situation. This demonstrates how well it works as a strong and dependable optimization tool for beamforming antenna arrays.

**Table 2:** Comparison of error between proposed approach against different optimization algorithms in different beam pattern.

		Optimization algorithms					
		ARO	GWO	PSO	WSO	BAE	CFO
Chebyshav array (N=12)	Min	6.13019915	6.251395	61.16075	13.17104815	6.336836892	6.042562647
	Max	11.02423267	117.8846	2226.043	852.1829112	515662273.6	6.050156608
	Average	7.236995703	14.11338	303.9005	169.8378615	35159417.52	6.044066032
	STD	1.317504191	24.92521	434.1903	188.8969327	120551997.4	0.002804153
Chebyshav array (N=13)	Min	0.231217195	0.920991	5.022827	13.79519617	0.22248078	0.039918736
	Max	4.463908386	23.05554	292.482	46.57937341	2452.273371	1.930211098
	Average	1.803982032	6.10548	130.6345	28.61069683	123.1936374	0.421248711
	STD	1.319405765	6.047123	78.62053	7.987001313	521.305342	0.620927878
Shaped array (N=12)	Min	2.769563547	3.882577	3.864313	10.60682771	3.02834662	2.728924258
	Max	3.9856684	17.71815	20.17152	28.9358839	26.04385001	9.758614099
	Average	3.372340888	6.703784	12.59201	18.23693544	10.44583402	3.372258787
	STD	0.319321033	5.063027	6.357597	5.32133212	7.992530039	2.06707335
Shaped array (N=13)	Min	0.125744614	0.136233	0.306727	1.546625793	0.102203786	0.055236892
	Max	3.185064767	0.389721	15.29302	33.35871546	21.52116413	2.915429789
	Average	0.933776938	0.234473	3.158597	12.47278158	6.77656265	1.784788838
	STD	1.172145806	0.070024	3.588398	8.282719189	6.975543965	1.334128339

#### Optimized inter-element spacing and beamwidth results

Tables 3, 4, 5 and  6 show the optimized inter-element spacing values for both Chebyshev and shaped arrays with varying numbers of antenna elements ($N=12$ and $N=13$) using the suggested CFO and benchmark methods. These findings show how each method distributes antenna parts to generate the desired beam patterns while minimizing sidelobe levels and system complexity. It is clear that the CFO algorithm provides more equal and balanced element spacings than other approaches, avoiding extreme or irregular distances that might lead to poor radiation properties. For example, in Tables 3 and 4 (Chebyshev arrays), CFO keeps element spacing near to the normal half-wavelength spacing, resulting in robust beam steering and excellent SLL suppression. Similarly, in Tables 5 and 6 (shaped arrays), CFO delivers compact and consistent element arrangements, but PSO and WSO frequently result in clustered or widely separated spacings. This balanced distribution immediately contributes to increased energy efficiency by reducing the number of active elements needed while maintaining array gain and beam directivity. Overall, the results show that CFO not only optimizes excitation coefficients but also assures that array designs are feasible and efficient enough for real-world applications.

**Table 3:** Inter-element spacing for Chebyshav beam pattern ($N=12$).

	Optimization algorithms					
n	ARO	GWO	PSO	WSO	BAE	CFO
1	0	0	0	0	0	0
2	0.82343	0.834618	0.9	0.825183	0.808623495	0.8403045
3	0.834706	0.836328	0.819693	0.856314	0.825899029	0.835989579
4	0.840852	0.843673	0.9	0.836566	0.842060714	0.843054516
5	0.849758	0.848925	0.844116	0.856314	0.848484813	0.849146959
6	0.850754	0.848412	0.860671	0.856314	0.851343564	0.851075952
7	0.851829	0.851099	0.858068	0.853571	0.852643296	0.851857465
8	0.851739	0.852314	0.861883	0.856314	0.852320178	0.850997931
9	0.848204	0.846226	0.856133	0.85138	0.850450524	0.848280875
10	0.845128	0.840697	0.833587	0.856314	0.845853534	0.843585123
11	0.83108	0.829237	0.830783	0.827486	0.840801373	0.829993362
12	0.842704	0.832841	0.760696	0.820124	0.85598667	0.826294179

**Table 4:** Inter-element spacing for Chebyshav beam pattern ($N=13$).

	Optimization algorithms					
n	ARO	GWO	PSO	WSO	BAE	CFO
1	0	0	0	0	0	0
2	0.705694	0.761026	0.815592	0.845568	0.730511851	0.652101842
3	0.756419	0.791574	0.822959	0.845568	0.770500961	0.730933642
4	0.788745	0.799391	0.832641	0.845568	0.792090165	0.780760825
5	0.808155	0.810819	0.833099	0.84553	0.801580454	0.805558557
6	0.818337	0.804498	0.812183	0.858722	0.806025449	0.818262672
7	0.820575	0.802698	0.712543	0.845568	0.806321014	0.824757972
8	0.822598	0.79321	0.5	0.844269	0.806052769	0.827283763
9	0.817165	0.787388	0.670033	0.845568	0.801692993	0.826740737
10	0.811454	0.770709	0.801445	0.845568	0.795293637	0.822485691
11	0.798041	0.765855	0.825418	0.811457	0.783555775	0.812338457
12	0.765013	0.744618	0.824463	0.82372	0.761946855	0.78857713
13	0.707605	0.739705	0.833011	0.845568	0.738954017	0.743060529

**Table 5:** Inter-element spacing for Shaped beam pattern ($N=12$).

	Optimization algorithms					
n	ARO	GWO	PSO	WSO	BAE	CFO
1	0.626268	0.72709	0.9	0.625336	0.676606342	0.537639272
2	0.67244	0.627595	0.662124	0.653332	0.666501284	0.672258627
3	0.764404	0.755331	0.76569	0.808328	0.769774714	0.763223656
4	0.698557	0.669731	0.682672	0.641765	0.692781355	0.700384406
5	0.598902	0.586128	0.599335	0.595289	0.602322934	0.599468204
6	0.643212	0.547449	0.5	0.606378	0.625952842	0.645640274
7	0.881685	0.580335	0.5	0.792035	0.779758758	0.899999674
8	0.546918	0.719766	0.812874	0.734141	0.51821147	0.551004205
9	0.531447	0.685662	0.745006	0.632364	0.62474739	0.510795187
10	0.698764	0.712987	0.699604	0.593902	0.714183084	0.698292798
11	0.893822	0.793315	0.9	0.75602	0.859085682	0.89999996
12	0.598383	0.806715	0.659831	0.693522	0.672585224	0.583357111

**Table 6:** Inter-element spacing for Shaped beam pattern ($N=13$).

	Optimization algorithms					
n	ARO	GWO	PSO	WSO	BAE	CFO
1	0.699946	0.524955	0.9	0.9	0.594576739	0.590710288
2	0.540763	0.557403	0.5	0.556314	0.528521253	0.532453217
3	0.623094	0.636536	0.622471	0.542681	0.630546039	0.651817857
4	0.647594	0.65048	0.696696	0.562537	0.658610361	0.686064304
5	0.553442	0.543357	0.584105	0.575804	0.561025042	0.569639436
6	0.545513	0.54587	0.603699	0.555884	0.550733839	0.578424203
7	0.557827	0.535704	0.5	0.625073	0.546487659	0.500105027
8	0.774524	0.738957	0.5	0.899998	0.734752153	0.611303337
9	0.637556	0.638702	0.736672	0.680152	0.633995978	0.680502133
10	0.61516	0.634134	0.703568	0.581987	0.625791388	0.664144091
11	0.666456	0.678646	0.694374	0.581717	0.671864255	0.677523351
12	0.824527	0.850088	0.9	0.806589	0.810531504	0.815052652
13	0.521848	0.501977	0.5	0.53463	0.565989056	0.549181007

Tables 7 and  8 show the excitation coefficients and phase of the beamwidth achieved with the CFO algorithm for both Chebyshev and shaped array configurations at $N=12$ and $N=13$. The results show that CFO can keep beamwidths relatively close to the desired standards, with few variances across varied antenna settings. The estimated beamwidths for the Chebyshev array remain steady, with extremely minimal imaginary components, showing that the CFO solutions are precise and numerically stable. The consistency is most noticeable at $N=13$, where beamwidth values are tightly grouped around 1.2-5.0, with minor error terms, indicating accurate control of the main lobe width. In contrast, the beamwidth values for the shaped array show greater fluctuations with real and imaginary components, which reflects the more difficult optimization requirements of shaped patterns. For both $N=12$ and $N=13$, CFO delivers very competitive results, with values generally falling within acceptable ranges and convergent toward stable averages. The comparatively minor variations noted, particularly in the N=13 scenario (beamwidth values ranging from 0.21 to 0.46), show how resilient CFO is at maintaining the required directivity when managing challenging array synthesis jobs.

**Table 7:** Excitation coefficients of the proposed caterpillar fungus optimization for different array antenna beam pattern.

n	Chebyshav array (N=12)	Chebyshav array (N=13)	Shaped array (N=12)	Shaped array (N=13)
1	1.392	1.119	0.4005	0.3647
2	1.917	1.379	0.6021	0.4669
3	2.949	2.268	0.7885	0.6494
4	3.962	3.25	1.1459	0.9285
5	4.763	4.153	1.1408	1.1304
6	5.201	4.811	0.6837	0.8893
7	5.186	5.094	0.3436	0.3701
8	4.726	4.944	0.2745	0.2941
9	3.916	4.391	0.3458	0.4069
10	2.927	3.546	0.2842	0.3453
11	1.927	2.566	0.1271	0.2519
12	1.385	1.611	0.2245	0.1081
13		1.223		0.2103

**Table 8:** Phase of the proposed caterpillar fungus optimization for different array antenna scenario of shaped array.

n	Shaped array (N=12)	Shaped array (N=13)
1	− 59.2233	− 69.4073
2	10.2193	1.379
3	52.742	42.2671
4	104.7718	78.9911
5	171.2301	140.0548
6	− 132.3579	− 164.6472
7	− 141.2177	− 109.3741
8	− 131.1844	− 147.6816
9	− 90.0839	− 127.2301
10	− 57.8401	− 82.7813
11	− 80.9162	− 45.3441
12	4.3116	− 80.6921
13		1.2342

#### Beamforming performance parameters

Table 9 compares the CFO method to ARO, GWO, PSO, WSO, and BAE in terms of key beamforming performance parameters such as SLL, beamwidth (BW), and statistical error indicators. The CFO algorithm outperforms standard references ($SLL_S$, $BW_S$) in terms of accuracy and error minimization across multiple circumstances.

For the Chebyshev array with N=12, CFO has an average error of 6.04, which is 16.5% lower than ARO, 57.2% lower than GWO, 98.0% lower than PSO, 96.4% lower than WSO, and nearly 100% lower than BAE. Similarly, the maximum error has been greatly decreased, demonstrating the resilience of the suggested strategy.

When N is increased to 13, CFO displays even more accuracy, with an average error of 0.42. This translates to gains of 76.6% over ARO, 93.1% over GWO, 99.7% over PSO, 98.5% over WSO, and 99.7% over BAE. In terms of SLL, CFO keeps values relatively close to the standard $SLL_S$, but rival algorithms have significant deviations, with PSO and WSO showing discrepancies of more than 5-10 dB.

For the shaped array with N=12, CFO obtains an average error of 3.37, which is comparable to ARO but has a 59.2% smaller standard deviation, showing greater stability. Compared to GWO (6.70), PSO (12.59), WSO (18.24), and BAE (10.44), the CFO improves by 49.7%, 73.2%, 81.5%, and 67.7%, respectively, indicating considerable advantages. The $SLL_{alg}$ achieved by CFO is nearly identical to the normal $SLL_S$, while other methods diverged significantly.

For the shaped array with N=13, CFO achieves an average error of 1.78, exceeding WSO (9.37) by 80.9%, PSO (6.52) by 72.7%, and BAE (6.75) by 73.6%. While ARO and GWO have somewhat lower averages in this scenario, CFO has the lowest minimum error (0.0552) and produces extremely consistent results, ensuring more reliable performance. Furthermore, CFO keeps beamwidth ($BW_{alg}$) within 1-2% of the conventional $BW_S$, whereas PSO and WSO cause deviations of more than 10%, resulting in decreased directivity.

**Table 9:** Parameters values for different optimization algorithms in different beam pattern.

	Algorithm	SLL_n	SLL_r	errorw	errorw_max	errorw_min	errorw_average	HPBW_n	HPBW_r
Chebyshav array (N=12)	ARO	− 29.0824	− 29.9845	6.13019915	0.032310364	1.18801E-08	0.008769956	6.187944	6.187944
	GWO	− 29.0974	− 29.9845	6.251395321	0.025169052	6.72719E-08	0.008943341	6.187944	6.187944
	PSO	− 26.3956	− 29.9845	61.1607487	0.472258101	1.42127E-07	0.087497495	6.187944	6.187944
	WSO	− 28.717	− 29.9845	13.17104815	0.123327068	5.29013E-09	0.018842701	6.187944	6.187944
	BAE	− 29.0777	− 29.9845	6.336836892	0.047609145	1.3834E-09	0.009065575	6.187944	6.187944
	CFO	− 29.0794	− 29.9845	6.042562647	0.027410675	1.82476E-08	0.008644582	6.187944	6.187944
Chebyshav array (N=13)	ARO	− 29.8504	− 29.9845	0.231217195	0.004309435	2.91106E-10	0.000330783	6.187944	6.187944
	GWO	− 29.6203	− 29.9845	0.920990546	0.012980949	1.40259E-09	0.001317583	6.187944	6.187944
	PSO	− 29.1697	− 29.9845	5.022826786	0.023914294	4.75264E-09	0.007185732	6.187944	6.187944
	WSO	− 28.4156	− 29.9845	13.79519617	0.161306068	1.91899E-07	0.019735617	6.187944	6.187944
	BAE	− 29.8077	− 29.9845	0.22248078	0.001075985	3.11243E-09	0.000318284	6.187944	6.187944
	CFO	− 29.9081	− 29.9845	0.039918736	0.000388354	1.22744E-09	5.71083E-05	6.187944	6.187944
Shaped array (N=12)	ARO	− 4.47331	− 5.15588	2.769563547	0.02682128	2.25781E-10	0.002766797	4.68	4.86
	GWO	− 3.85862	− 5.15588	3.882577071	0.031890067	4.68385E-09	0.003878698	4.59	4.86
	PSO	− 3.1919	− 5.15588	3.864312875	0.030030382	4.79046E-09	0.003860452	4.77	4.86
	WSO	− 2.62229	− 5.15588	10.60682771	0.111030331	3.16044E-09	0.010596231	4.68	4.86
	BAE	− 4.09852	− 5.15588	3.02834662	0.025938056	6.68488E-09	0.003025321	4.59	4.86
	CFO	− 4.57166	− 5.15588	2.728924258	0.027411073	5.47886E-09	0.002726198	4.68	4.86
Shaped array (N=13)	ARO	− 5.35397	− 5.15588	0.125744614	0.002031798	5.97218E-10	0.000125619	4.86	4.86
	GWO	− 5.46201	− 5.15588	0.136232503	0.002821507	4.55817E-11	0.000136096	4.77	4.86
	PSO	− 5.69568	− 5.15588	0.306726927	0.002686362	9.9468E-12	0.000306421	4.77	4.86
	WSO	− 4.31248	− 5.15588	1.546625793	0.019496727	3.82417E-10	0.001545081	4.86	4.86
	BAE	− 5.43577	− 5.15588	0.102203786	0.000665935	7.71311E-11	0.000102102	4.86	4.86
	CFO	− 5.42925	− 5.15588	0.055236892	0.000283559	1.18628E-11	5.51817E-05	4.86	4.86

For the shaped-array instance with $N=12$, CFO obtained an SLL of $-4.57$ dB compared to the reference value of $-5.16$ dB, resulting in a moderate divergence of roughly 0.59 dB. This difference can be attributed to the lower number of antenna elements, which reduces the degrees of freedom available for correctly designing the radiation pattern. With fewer parts, the optimization process becomes more limited, making it more difficult to achieve many objectives concurrently, such as tight sidelobe suppression and beamwidth preservation. As a result, lower-dimensional configurations should see some performance reduction in SLL.

On the other hand, in the $N=13$ situation, the extra antenna element offers more control over the amplitude and phase distribution throughout the array, allowing for better sidelobe suppression and a more accurate approximation of the desired pattern. This draws attention to a crucial trade-off between system complexity and performance: while fewer antenna elements impose stricter constraints that may result in slight departures from the ideal response, more antenna elements improve the solution space and enable optimization algorithms like CFO to achieve better radiation characteristics.

#### Convergence rate

**Fig. 11:**
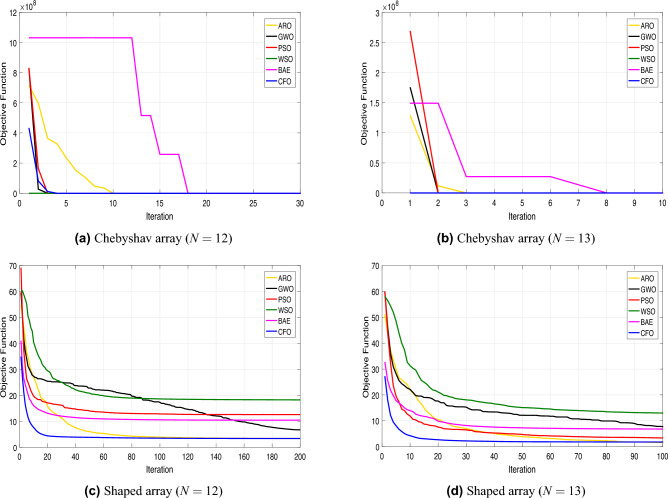
Average convergence rates of different optimization algorithm.

Figure 11 illustrates the average convergence behavior of the comparing optimization techniques for shaped-array synthesis with $N=12$ and $N=13$. The CFO achieves the fastest reduction in the objective function during the first 40 iterations before eventually approaching a stable minimum with a smooth convergence curve. This tendency can be related to CFO’s balanced search mechanism, in which the wave advance operator improves global exploration during the first iterations, allowing the algorithm to quickly find interesting sections of the search space. Subsequently, the spiral rising and parasitic behavior operators accelerate the local exploitation process, leading to the gradual refining of the best candidate solutions. Such synchronized transitions between exploration and exploitation avoid sudden oscillations in fitness levels and prevent premature stagnation, resulting in smooth and steady convergence curves for CFO.

In contrast, algorithms such as GWO and WSO have a slower fitness reduction since their search process requires more iterations to balance diversification and intensification. PSO converges swiftly at first, but flattens earlier, indicating a larger likelihood of trapping in local optima. ARO and BAE have moderate convergence rates but require more iterations to achieve competitive objective values. CFO’s excellent convergence trend supports its capacity to efficiently navigate the nonlinear beamforming optimization terrain while retaining stable search behavior.

In the $N=13$ Chebyshev case, despite a higher dimensional search space compared to $N=12$, convergence occurred in fewer iterations. This is attributed to the geometry of the optimization landscape and the additional antenna element that enhances flexibility in excitation distribution, leading to smoother feasible regions. This allows for quicker satisfaction of sidelobe and beamwidth constraints. Additionally, odd-element Chebyshev arrays often exhibit more symmetric excitation structures, facilitating earlier attainment of high-quality solutions during the search process. In the $N=12$ case, limited degrees of freedom complicate achieving the desired radiation characteristics, leading to narrower feasible regions and stronger variable coupling. This results in slower convergence, which reflects the heightened optimization challenge rather than inferior scalability.

#### Performance stability and variability evaluation

**Fig. 12:**
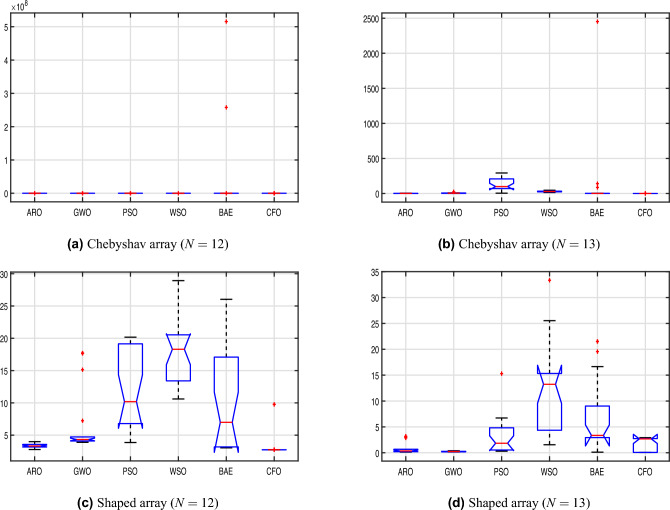
Box plot of the objective functions obtained by different optimization algorithms over twenty runs.

Figure 12 shows a boxplot examination of the objective function values obtained by the CFO method when compared to ARO, GWO, PSO, WSO, and BAE. The findings shed light on the stability, robustness, and variability of each method across 20 independent runs. As shown, CFO has the smallest interquartile range (IQR) with very small whiskers, showing highly consistent performance with little change between runs. In contrast, PSO and WSO have large IQRs and multiple outliers, indicating poor stability and a significant sensitivity to beginning conditions. GWO and ARO achieve considerable consistency but still have greater spreads than CFO, but BAE exhibits significant variability with several distant outliers, indicating its unreliability for beamforming optimization.

The boxplot and statistical comparison results give a better picture of the optimization techniques’ robustness and repeatability across numerous separate runs. A tight interquartile range and a small overall spread suggest that the algorithm consistently converges to comparable solutions, indicating high stability and minimal susceptibility to random initialization. Wider distributions, or the existence of many outliers, indicate inconsistent search behavior and a larger reliance on stochastic elements. Furthermore, lower median goal values equate to better average optimization performance, demonstrating that the algorithm consistently produces high-quality beamforming designs. As a result, our statistical findings directly support the practical goal of getting consistent, repeatable, and high-performance antenna synthesis solutions rather than rare best-case outcomes.

### Wilcoxon and Friedman tests

Table 10 summarizes the statistics analysis of the A 12 Chebyscheff antenna case. The Friedman test yielded a highly significant *p*-value of $5.097\times 10^{-18}$, indicating obvious statistical differences among all algorithms evaluated. The CFO received the highest mean score of 1.00, indicating the strongest overall performance across all independent runs. Furthermore, the Wilcoxon signed-rank test demonstrates that CFO outperformed ARO, GWO, PSO, WSO, and BAE in all pairwise comparisons at the 0.05 significance level. CFO had a low variance of $7.863273\times 10^{-6}$ and the lowest coefficient of variation (0.0005), indicating stable and repeatable behavior. Competing approaches, particularly BAE and PSO, have significantly larger variances and confidence intervals, indicating unstable optimization behavior. The small bootstrap confidence interval of CFO ($[6.0431,\,6.0453]$) confirms its strong consistency and exceptional convergence dependability.

**Table 10:** Statistical analysis results for 12 Chebyscheff Antenna.

Algorithm	Mean rank	Variance	CV	95% Bootstrap CI	Wilcoxon vs CFO (*p*-value)
ARO	2.55	1.735817	0.1821	$[6.7543,\;7.8197]$	$4.010\times 10^{-5}$
GWO	2.64	$6.212663\times 10^{2}$	1.7661	$[6.9715,\;26.0427]$	$4.010\times 10^{-5}$
PSO	5.36	$1.885212\times 10^{5}$	1.4287	$[191.2240,\;499.8598]$	$4.010\times 10^{-5}$
WSO	4.64	$3.568205\times 10^{4}$	1.1122	$[102.9307,\;255.1160]$	$4.010\times 10^{-5}$
BAE	4.82	$1.453278\times 10^{16}$	3.4287	$[397.3537,\;9.3757\times 10^{7}]$	$4.010\times 10^{-5}$
CFO	1.00	$7.863273\times 10^{-6}$	0.0005	$[6.0431,\;6.0453]$	–

*Friedman Test*: *p*-value $=5.097\times 10^{-18}$

Table 11 shows the statistics analysis of the 13 Chebyscheff antenna example. The Friedman test resulted in a *p*-value of $5.484\times 10^{-16}$, indicating statistically significant performance differences amongst the evaluated algorithms. The CFO had the best mean rank of 1.41, followed by ARO (2.45) and BAE (3.00), suggesting higher overall optimization quality. In addition, the Wilcoxon signed-rank test shows that CFO surpassed all benchmark algorithms in pairwise comparisons. In terms of robustness, CFO had a low variance of $3.855514\times 10^{-1}$ and a confidence interval of $[0.1921,\,0.7034]$, indicating steady and reliable performance. Despite exhibiting moderate volatility, ARO’s mean objective values remained lower than CFO. Other algorithms, such as PSO, WSO, and BAE, showed significantly higher variances and broader confidence intervals, indicating less consistent search behavior. These data demonstrate that CFO is the best competitive and statistically proven solution to the Chebyscheff synthesis scenario.

**Table 11:** Statistical analysis results for 13 Chebyscheff Antenna.

Algorithm	Mean rank	Variance	CV	95% Bootstrap CI	Wilcoxon vs CFO (*p*-value)
ARO	2.45	1.740832	0.7314	$[1.2811,\;2.3437]$	$2.155\times 10^{-3}$
GWO	3.45	$3.656770\times 10^{1}$	0.9904	$[3.9478,\;8.8408]$	$6.978\times 10^{-5}$
PSO	5.77	$6.181187\times 10^{3}$	0.6018	$[100.2830,\;164.3467]$	$4.010\times 10^{-5}$
WSO	4.91	$6.379219\times 10^{1}$	0.2792	$[25.3685,\;31.9130]$	$4.010\times 10^{-5}$
BAE	3.00	$2.717593\times 10^{5}$	4.2316	$[1.6962,\;352.2889]$	$2.947\times 10^{-4}$
CFO	1.41	$3.855514\times 10^{-1}$	1.4740	$[0.1921,\;0.7034]$	–

*Friedman Test*: *p*-value $=5.484\times 10^{-16}$

### Computational complexity analysis

The computational complexity of the compared population-based optimization methods is mainly determined by the population size *N*, the maximum number of iterations $Max_T$, and the computing cost of the fitness evaluation $F_i$. The majority of benchmark methods, such as ARO, GWO, PSO, and WSO, have a temporal complexity of $\mathcal {O}(N \times Max_T \times F_i)$. In comparison, BAE and CFO require additional internal updating procedures, resulting in significantly greater computing burdens of $\mathcal {O}(2.2 \times N \times Max_T \times F_i)$ and $\mathcal {O}(2 \times N \times Max_T \times F_i)$.

To provide a practical evaluation beyond theoretical complexity, all algorithms’ runtime and memory consumption were assessed under identical simulation settings, ensuring a fair comparison. The results are summarized in Table 12.

**Table 12:** Computational complexity comparison with runtime and memory usage.

Algorithm	Big $\mathcal {O}$	Elapsed time per iteration (s)	Memory usage per iteration (MB)
ARO	$\mathcal {O}(N \times Max_T \times F_i)$	1.2612	3194
GWO	$\mathcal {O}(N \times Max_T \times F_i)$	1.1874	3132
PSO	$\mathcal {O}(N \times Max_T \times F_i)$	0.9288	3183
WSO	$\mathcal {O}(N \times Max_T \times F_i)$	1.3627	3199
BAE	$\mathcal {O}(2.2 \times N \times Max_T \times F_i)$	1.7006	3158
CFO	$\mathcal {O}(2 \times N \times Max_T \times F_i)$	1.5070	3108

As demonstrated in Table 12, PSO achieved the lowest execution time per iteration, followed by GWO and ARO. The CFO required a little longer runtime than some benchmark techniques due of the additional exploration and exploitation operators. However, CFO had the lowest memory use (3108 MB) of all studied algorithms, demonstrating efficient storage utilization during optimization.

### Performance analysis of non-dominant CFO cases

Even while the CFO performed the best overall in most of the benchmark situations that were taken into consideration, there were a few instances where rival algorithms yielded results that were either significantly better or equivalent. In order to offer a fair and thorough interpretation of the numerical results, these cases are explored below. Sometimes, techniques like GWO and ARO produced slightly lower goal values than CFO in some low-dimensional optimization problems. This tendency is mostly explained by the more straightforward search environment, which allows quick exploitation-oriented methods to converge effectively without requiring a lot of investigation. Similar to this, in certain Chebyscheff synthesis scenarios, the desired radiation pattern has symmetric and regular features that allow traditional swarm-based approaches to quickly get close to near-optimal solutions.

Additionally, certain convergence curves indicate that PSO or GWO reduced fitness more quickly in the initial iterations. This rapid initial progress is typically caused by aggressive leader-following or velocity-driven updates that favor short-term exploitation. However, CFO maintains progressive improvement through its balanced exploration and exploitation operations, while conventional approaches may eventually suffer from stagnation or decreased population variety. CFO did not consistently record the minimal variation in a small number of robustness comparisons. In these situations, certain algorithms rapidly converged on small regions, producing statistical spreads that were narrower but not necessarily better global solutions.

In certain test situations, GWO occasionally scored better mean ranks for shaped-array synthesis challenges. This could happen if there are smooth local basins in the optimization landscape where hierarchical encircling behavior works especially well. However, CFO continued to show consistent performance over several runs and remained fiercely competitive in these situations. .

## Conclusion

In this paper, we develop a novel beamforming optimization approach based on the CFO algorithm for antenna array synthesis in beyond 5G and future 6G wireless communication systems. The major goal was to improve spectral efficiency, reduce SLL, and increase energy economy by optimizing inter-element spacing and excitation management. A detailed comparison research is carried out against five benchmark algorithms (ARO, WSO, GWO, PSO, and BAE) in both Chebyshev and shaped array configuration. The simulation results indicated that the CFO algorithm regularly outperformed these benchmark approaches in terms of accuracy, stability, and convergence speed. CFO achieved much lower minimum, maximum, and average error levels, created SLL values closer to the intended standard, and kept beamwidths as near to reference values as possible. Statistical results and boxplot studies showed that CFO ensures low variability, strong convergence, and consistent performance across several runs. These gains were realized with no added computing complexity, making CFO an excellent choice for large-scale antenna array optimization.

Looking ahead, this work can have an even greater influence in a number of future research topics. The creation of multi-objective CFO variations that may concurrently optimize trade-offs between communication security, energy consumption, and spectrum efficiency is one possible approach. In order to improve adaptability in dynamic contexts and speed up convergence, CFO can also be integrated with hybrid optimization frameworks, such as by combining it with deep learning-based techniques or classical approaches. Furthermore, using CFO with cutting-edge architectures like fluid antenna system (FAS) and Reconfigurable Intelligent Surface (RIS) may help solve new problems in networks that go beyond 5G and 6G. CFO-based beamforming’s hardware and real-time implementations are crucial steps in confirming its viability for massive MIMO systems.

## Data Availability

The datasets used and/or analysed during the current study available from the corresponding author on reasonable request.
